# Impact of a 5-Year Mass Drug Administration Programme for Soil-Transmitted Helminthiases on the Spatial Distribution of Childhood Anaemia in Burundi from 2007 to 2011

**DOI:** 10.3390/tropicalmed7100307

**Published:** 2022-10-17

**Authors:** Mohamad Assoum, Giuseppina Ortu, Maria-Gloria Basáñez, Colleen Lau, Archie C. A. Clements, Kate Halton, Alan Fenwick, Ricardo J. Soares Magalhães

**Affiliations:** 1 Children’s Health and Environment Program, Child Health Research Centre, The University of Queensland, Brisbane, QLD 4101, Australia; 2 UQ Spatial Epidemiology Laboratory, School of Veterinary Science, The University of Queensland, Via Warrego Highway, Gatton, QLD 4343, Australia; 3 School of Medicine, The University of Queensland, Brisbane, QLD 4006, Australia; 4 Schistosomiasis Control Initiative, Department of Infectious Disease Epidemiology, School of Public Health, Faculty of Medicine (St. Mary’s Campus), Imperial College London, Norfolk Place, London W2 1PG, UK; 5 London Centre for Neglected Tropical Disease Research and MRC Centre for Global Infectious Disease Analysis, Department of Infectious Disease Epidemiology, School of Public Health, Imperial College London, Norfolk Place, London W2 1PG, UK; 6 Research School of Population Health, Australian National University, Canberra, ACT 2601, Australia; 7 Institute of Health and Biomedical Innovations, Queensland University of Technology, Brisbane, QLD 4059, Australia

**Keywords:** soil-transmitted helminthiases, *Ascaris lumbricoides*, *Trichuris trichiura*, hookworm infection, anaemia, school-aged children, model-based geostatistics, spatiotemporal modelling, predictive risk mapping

## Abstract

Background: Childhood anaemia affects 1.8 billion people globally. Little is known about the long-term impact of mass drug administration (MDA) for the control of soil-transmitted helminthiases (STH) on the spatiotemporal variation of anaemia prevalence and severity. We describe the long-term spatiotemporal impact of a 5-year STH MDA programme (2007–2011) on the prevalence of anaemia and anaemia severity in school-aged children (SAC) in Burundi. Methodology/Principal Findings: We used annual haemoglobin concentration and STH data collected during 2007–2011 in 31 schools in Burundi. Spatial dependence in prevalence and severity of anaemia was assessed using semivariograms. Bayesian geostatistical models were developed to (a) quantify the role of STH (adjusted for other anaemia determinants) in the spatiotemporal distribution of anaemia prevalence/severity, and (b) predict the geographical variation of both outcomes across Burundi. Adjusted population data were used to estimate the geographical distribution of the number of SAC at risk of anaemia and with low and moderate/severe anaemia. Infections with *Ascaris lumbricoides* and *Trichuris trichiura* were positively and significantly associated with childhood anaemia; hookworm infections were not. A significant decrease in anaemia prevalence, from 40–50% (2008) to 10–20% (2011) was predicted in western areas. The predicted prevalence of low-severity anaemia decreased from 40–50% (2008) to <20% (2011) in southern and eastern areas. Moderate/high-severity anaemia was concentrated in western regions of Burundi, with pockets of moderate/high-severity anaemia in central and northern regions in 2008. The overall number of predicted anaemic children decreased from 443,657 (2008) to 232,304 (2011), with a resurgence after MDA disruption in 2010 (to 480,605). Prevalence of low- and moderate-severity anaemia was higher in boys than in girls. Conclusions/Significance: Despite ongoing MDA, the prevalence of anaemia in SAC remained high and increased in certain parts of the country. It is recommended that MDA programmes targeting STH are complemented with specific anaemia interventions.

## 1. Introduction 

Anaemia affects 1.8 billion people globally [1]. In children, anaemia can cause impaired cognitive development, compromised immune system development, and increased risk of mortality [2]. Several risk factors are associated with anaemia including individual, household and environmental factors. Main individual factors are infectious diseases and genetic factors. Infectious diseases include human immunodeficiency virus (HIV), malaria and neglected tropical diseases such as soil-transmitted helminthiases (STH) and schistosomiasis [3]. Genetic factors include inherited blood disorders such as sickle cell anaemia and thalassaemia [4,5,6]. Household factors involve socioeconomic status of the household, poverty and associated individual-level factors such as malnutrition, low sanitation and poor hygiene practices [1,6,7,8]. Though little is known about the more distal environmental factors, there is an established connection between anaemia and landcover/land use, access to fresh water, precipitation and elevation, which modulate other proximal individual-level and household factors [9,10].

Anaemia is associated with STH (*Ascaris lumbricoides*, *Trichuris trichiura* and hookworm infections (*Necator americanus* and *Ancylostoma duodenale*)), as the presence of these parasites leads to occult bleeding through the bowel, anaemia of inflammation and reduction in available macro- and micronutrients within the host’s body [11]. Prevalence of hookworm has long been associated with the prevalence of anaemia [5,9,12,13,14,15,16]. Numerous studies have examined the relationship between anaemia, STH and other environmental and socioeconomic covariates [5,7,8,12,13,15,16,17,18,19]. These studies highlight a positive correlation between hookworm infections, poor socioeconomic standing, low vegetation cover and high prevalence of anaemia.

For the past decade, the World Health Organization (WHO) has adopted Mass Drug Administration (MDA) deworming programmes as their key STH control intervention to target and manage STH-related co-morbidities, including nutritional deficiencies and poor growth outcomes [20]. Following MDA, treated children were found to have better growth outcomes than those untreated. Whilst the effects of MDA on blood haemoglobin (Hb) were found to be directly non-significant, significantly improved appetites and improved physical growth outcomes were observed in the treated population. This improvement also brought about an indirect improvement in blood Hb levels [21]. This was demonstrated over many geographies as demonstrated by Hall et al. 2008 which was a meta-analysis summarizing the effects over multiple studies [21].

In Burundi, anaemia is a major health issue, with an estimated 3 million, out of 10 million people being anaemic [1], of which up to 47% are school-aged children (SAC) [1,22]. Since 2007, control of STH and schistosomiasis has been implemented via MDA programmes conducted by the Schistosomiasis Control Initiative (SCI) [23] with the aim of reducing the impact of co-morbidities, in particular anaemia [24]. A history of political and social instability has caused disruptions of treatments in 2010 and from 2015 to 2020, resulting in MDA coverage <50% in 2010 and limited and varying coverage from 2015 onwards. The MDA programme delivery of albendazole was expected to reduce the overall prevalence of anaemia in Burundi by reducing the prevalence of *A. lumbricoides*, *T. trichiura* and hookworm infections. Thus, the disturbance of the programmes, which resulted in a resurgence of these parasites [24,25], is also expected to have led to an increase in anaemia prevalence.

Previous studies in West Africa have developed predictive anaemia prevalence and anaemia severity maps using ecological relationships with STH and environmental predictors [26,27], and found significant relationships between hookworm infection, land surface temperature, normalized difference vegetation index (NDVI) and anaemia prevalence.

While relationships between STH and anaemia prevalence are assumed, these assumptions have not been tested by monitoring anaemia rates during long-standing MDA programmes, nor have current studies made use of individual-level and temporally measured data on STH and anthropometric measurements to develop predictive anaemia prevalence and anaemia severity maps. Spatiotemporal modelling of anaemia can help to monitor and evaluate the impact of public health interventions such as MDA programmes for STH, as well as guide future interventions to reflect the distribution of populations most at risk.

Therefore, this study aimed to (a) evaluate the spatial variation in the impact of a 5-year STH MDA programme on prevalence of anaemia and anaemia severity in SAC; (b) identify communities where the estimated total number of anaemic children is at its highest, and (c) evaluate the impact of MDA programme disruption on the anaemia indicators investigated and the number of anaemic SAC in Burundi. 

## 2. Materials and Methods

### 2.1. Ethical Considerations 

Ethical clearance was obtained from the Research Ethics Committee of Imperial College London (ICREC_8_2_2) and by the Ministry of Health in Burundi. Verbal consent were provided by teachers on behalf of parents, and by the enrolled children. For all surveys, individual child names were registered on paper forms to ensure unique sample collection and individual anthelmintic treatment if necessary. Those found positive for STH at the time of surveys received anthelmintic treatment immediately, but children found to be anaemic were not provided with any specific treatment for anaemia. In addition, MDA rounds were provided to all schools, including schools which were not part of the sentinel sites, two to four weeks after surveys were conducted. Children’s names were not entered into the database; instead the original paper forms were stored by the Burundian Ministry of Health. Data were anonymized prior to analysis by assigning each participant a unique identification number. 

### 2.2. Data Collection on Anaemia and Soil-Transmitted Helminthiases

The protocol for data collection for the 2007–2011 school-based surveys has been reported elsewhere [28]. In brief, the surveys were conducted in conjunction with the delivery of an STH MDA programme in each school targeting children aged from 5 to 15 yrs. Data were gathered and collected from the 12 pilot sites in 2007 and the same 12 pilot sites in addition to 19 extension schools (31 schools in total) during three years (2008, 2009 and 2011); however, due to civil unrest, only the initial 12 pilot sites out of the 31 were surveyed in 2010. Data collection and sampling methods have been described in detail elsewhere [24,25]. A total of 40,656 children were sampled over the 5 years and data on each child included age, sex, blood Hb levels, egg counts for *A. lumbricoides*, *T. trichiura* and hookworms, height, weight, and school location.

Between 2007 and 2010 each child was tested for anaemia using HemoCue strips, which provide a quantitative measurement of Hb concentration [29]; in 2011 children were tested for Hb concentration using the Lovibond blood test, which provides a colour-based qualitative measurement (21). All Hb results were adjusted for elevation above sea level [30,31]. To allow comparability with HemoCue results from 2007–2010, the 2011 Hb Lovibond test results were adjusted by multiplying by a factor of 1.14 to adjust for reader error [32]. In order to determine the prevalence of anaemia and anaemia severity, Hb levels were categorised using WHO guidelines based on age and sex as shown in Table S1 [33].

Micro-blood samples were taken from 100 children (aiming for 50 boys and 50 girls) per school using HemoCue strips. Each year, samples were collected in May and the MDA round was delivered in June. 

Height and weight measurements were collected for every child and used to calculate anthropometric Z-scores for each child. Weight-for-Age (WAZ), and Height-for-Weight are predominately used for children under the age of 5; our study population ranged from 5 to 15 yrs and to have a consistent measure of malnutrition across all ages we calculated Height-for-age Z-scores (HAZ) for every child using the WHO AnthroPlus tool [34]. However, due to data limitations, not every child had a WAZ and HAZ.

Geographical coordinates of each school were recorded using hand-held global positioning system (GPS) units and summary data were plotted in a geographical information system (GIS) (ArcMap version 10.3, ESRI, Redlands, CA, USA). 

### 2.3. Environmental, Infection and Population Data

Data on socioeconomic status (SES) of households per district of Burundi were obtained from the Burundi Demographic Health Survey (DHS) of 2010 using the household wealth index. SES results were analysed on a continuous scale and as such were standardised using the arithmetic mean and standard deviation. Using interpolation methods, Inverse Distance Weighted (IDW), in ArcGIS, SES was predicted across the country using 1 km by 1 km grids. For modelling purposes, SES of school sites were used for children as it was not possible to identify the SES of children’s homes.

Malaria prevalence for 2007–2011 were obtained using 5 km by 5 km predicted prevalence maps from the Malaria Atlas Project (MAP) [35,36]. Due to gaps in the MAP maps, focal statistics/nearest neighbour methods in ArcGIS were used to fill in gaps for the prevalence maps per year. For modelling purposes, predicted malaria prevalence values were extracted for each school location using the GIS, because the children were not tested for malaria in this survey.

Electronic data for normalised difference vegetation index (NDVI) for a 30 m × 30 m grid cell resolution were obtained from Landsat 5 and 8 satellite images via the Google Earth Engine database. The locations of large perennial inland water bodies were obtained from the Food and Agriculture Organization of the United Nations [37] and the distance to perennial inland water bodies (DPWB) were estimated for each survey location using GIS. Elevation data with a grid resolution of 30 m × 30 m, was generated by a digital elevation model from the Advanced Space-borne Thermal Emission and Reflection Radiometer (ASTER) Global Digital Elevation Model (GDEM) were used. Land surface temperature (LST) data with a 500 m × 500 m resolution were also obtained from the ASTER system. WorldClim generated precipitation data with 1 km × 1 km grid resolution were also obtained. Monthly remotely sensed data for LST and NDVI from 2007 to 2011 were used to create a new annual raster file. A population surface map that was derived from the Global Rural-Urban Mapping Project (GRUMP) beta product with a 5 km × 5 km resolution was obtained from the Centre for International Earth Science Information Network (CIESIN) of the Earth Institute at Columbia University, New York [38]. GIS was used to extract the values at each survey location (i.e., school) for all environmental datasets and not at individual child home locations. However, for LST, NDVI and precipitation, only maps from 2011 were used as little variance was evident between maps for each year.

Predictive prevalence maps for *A. lumbricoides*, *T. trichiura* and hookworms developed in our previous study [25], with approximately 30 m × 30 m cell size, were used in the prediction phase of spatial modelling. 

### 2.4. Statistical Analyses 

#### 2.4.1. Non-Spatial Models of Prevalence of Anaemia and Anaemia Severity

Statistical analyses were conducted for two separate outcomes, namely, prevalence of anaemia (proportion of children found anaemic) and prevalence of anaemia severity (classed as the proportion of children non-anaemic, or with low, moderate or severe anaemia according to the WHO classification described in Supplementary Materials, Table S1). The relationships between each of the outcomes and all environmental, socioeconomic, anthropometric and infection covariates at each of the 31 sentinel school sites were investigated using scatterplots and best-fit lines. If the scatterplot and best-fit lines were found to be linear, the covariate was included in the univariable and multivariable models as a fixed effect, otherwise, a quadratic term for that variable was included in the univariable and multivariable models. Relationships between STH and anaemia prevalence were assessed using modified Rao-Scott chi-squared tests in STATA 13 (STATA Corporation, College Station, TX, USA). A modified Rao-Scott chi-squared test was selected as it tests for the likelihood ratio between anaemia prevalence and STH prevalence rather than the differences in proportions that a standard chi-squared test examines. The test was modified to adjust for clustering by site.

Fixed-effect binomial logistic regression models of presence of anaemia and multinomial logistic regression models of anaemia severity were developed in STATA version 13. Due to data distribution, particularly the small counts in the high anaemia severity category, the data for moderate- and high-severity anaemia were combined into a single category for the analysis of anaemia severity. All univariable models included the individual-level variables of age, sex and year of survey as fixed effects. Infection covariates included prevalence of *A. lumbricoides*, *T. trichiura*, hookworm and predicted prevalence of malaria with a quadratic transformation. Prevalence of STH co-infections and intensity of STH were not included in the analysis because prevalence of co-infections and moderate and high intensity infections were very low from 2008 onwards [24]. Environmental covariates included fixed effects for NDVI, LST and elevation, and quadratic transformations for DPWB and precipitation. Socioeconomic covariates included standardised socioeconomic scores only. Anthropometric covariates included standardised HAZ scores only. Variables included in the multivariable models were grouped into four categories. The first one comprised all fixed-effect variables, the second one the infection variables, the third one included the environmental variables and the fourth the anthropometric variables. All selected variables have a known correlation with anaemia prevalence and anaemia severity. 

Three tiered nested multivariable regression models were constructed to observe the impact of each group of covariates on the relationship with anaemia prevalence: (1) Model 1 included age and sex as fixed effects, survey year and (STH, malaria) infection as covariates; (2) Model 2 included the variables in Model 1 plus the environmental covariates (including SES); (3) Model 3 included all variables in Model 2 plus anthropometric/nutrition status (HAZ) covariates. All variables were included in the final multivariable model and adjusted for clustering effects.

#### 2.4.2. Analysis of Residual Spatial Dependence

Residual semivariograms from the final multivariable models for the prevalence of anaemia for each survey year were estimated using the geoR package of the statistical software R (The R Project for Statistical Computing, https://www.r-project.org/, accessed 30 June 2015). Semivariograms allow for the quantification of spatial clustering through the estimates of four key parameters: (i) the sill, which details the total variation; (ii) the partial sill (the difference between the nugget and the sill), which details the variation due to geographical variation; (iii) the nugget, which represents the variation due to non- spatially constructed factors, and (iv) the range, which represents the size of clusters of the variable in question (in our case anaemia prevalence) in decimal degrees. The proportion of variance of the data that can be attributed to spatial location was estimated by dividing the partial sill by the sill. When the sill of residual semivariograms is not within a reasonable range, this indicates the presence of first-order spatial variation, also termed spatial trend.

#### 2.4.3. Parameter Estimation, Spatial Risk Prediction and Model Validation

It was not possible to prepare anaemia maps for 2007 due to lack of data for that year. Parameter estimation and spatial prediction of prevalence of anaemia and of anaemia severity classes were performed for each year using, respectively, binomial model-based geostatistics (MBG) and multinomial MBG, within the Bayesian statistical software, OpenBUGS version 1.4 (Medical Research Council Biostatistics Unit, Cambridge, UK and Imperial College London, London, UK). For parameter estimation in both models, an initial model learning phase was conducted including year of survey, demographics, STH status, and anthropometric, environmental and socioeconomic data as fixed effects, and a geostatistical random effect, in which spatial autocorrelation between locations was modelled using an exponentially decaying autocorrelation function. Parameter *Φ* denotes the rate of decay of spatial autocorrelation, which was used to calculate the size of clusters, with 3/*Φ* providing cluster size in decimal degrees, and 1 decimal degree being equivalent to approximately 111.32 km [27]. In the spatial prediction phase, models included age, survey year, sex and a set of predicted values based on existing maps for predicted prevalence of STH (*A. lumbricoides*, *T. trichiura* and hookworms) [25], as well as the predicted prevalence of malaria [36].

The output of MBG models are called posterior distributions, from which the mean and the uncertainty associated with parameter estimates can be derived (full model in annex Figures S4–S6). Predicted values of anaemia prevalence and severity were summarised into the posterior mean, 95% credible intervals (95% CI) and standard deviation. Estimation of changes in the predicted surface area of the prevalence of anaemia and of anaemia severity categories was conducted in ArcGIS using its raster calculator and zonal statistics tools.

The accuracy of predictions of the prevalence of anaemia and prevalence of anaemia severity classes was assessed using the mean prediction error and the mean absolute error between the predicted and the observed values taken using a subsample of the same dataset to predict against [26,39]. The mean error quantifies the bias of the predictor, and the mean absolute error provides a measure of the association between the observed and the predicted values [26,39]. 

#### 2.4.4. Estimation of Number of School-Age Children at Risk of Anaemia

In order to quantify the burden of anaemia in Burundi (defined as the number of anaemic SAC per district in Burundi), population density maps were multiplied by the predicted prevalence of anaemia maps using ArcGIS version 10.5 (ESRI, Redlands, CA, USA). To capture changes in population growth rates, the population density maps were multiplied by the population growth rate from 2008 to 2011. The produced maps estimated the number of anaemic children per district as well as the number of children at risk of different anaemia severity classes.

## 3. Results

### 3.1. Dataset for Analysis

From 2008 to 2011, a total of 37,675 individual observations for Hb concentration across 31 schools (with the exception of 2010, when only 12 sites were surveyed), were included in the analysis. Seventeen percent of anaemic children were infected with *A. lumbricoides*, which was statistically significantly higher than in non-anaemic children (11%) (*p* < 0.001); in contrast, 8.8% of anaemic children were infected with hookworm, and this result was not significantly different from that in non-anaemic children (7.5%) (*p* = 0.1) (Table 1).

Infection with *T. trichiura* was more common in anaemic than in non-anaemic children, and this difference was statistically significant (6.3% vs. 4.4%, *p* = 0.03). The proportion of children with low-severity anaemia was higher in those infected with *A. lumbricoides* (15.1%), as well as in those infected with *T. trichiura* (6.3%). Whilst the proportion of children with low-severity anaemia was also high in children infected with hookworm (9.1%), this relationship was not statistically significant. The prevalence of moderate- and high-severity anaemia was found to be highest in children infected with *A. lumbricoides* (19.2% and 23%, respectively). The proportion of moderate- and high-severity anaemia was, respectively 8.5% and 8.1% in children with hookworm, and 5.1% and 15.1% in children with *T. trichiura* (Table 2).

### 3.2. Non-Spatial Statistical Modelling

In non-spatial regression models (Models 1–3), after adjusting for other covariates, the presence of anaemia, as a binary variable, was positively and statistically significantly associated with *A. lumbricoides* and *T*. *trichiura* infections (Odds Ratio (OR) = 1.56 and OR = 1.39, *p* < 0.0001 and 0.001, respectively). Prevalence of anaemia was not significantly associated with hookworm infection (OR = 1.09, *p* = 0.4). The prevalence of anaemia was negatively but not statistically significantly associated with malaria or SES (OR =0.96 and OR = 0.99 and *p* = 0.74 and 0.77, respectively). Of all environmental co-variates, only NDVI was significantly and negatively associated with the prevalence of anaemia (OR = 0.90, *p* < 0.0001). DPWB and elevation were both significantly and positively associated with the prevalence of anaemia (OR = 1.16 and OR = 1.63, *p* = 0.05, 0.03, respectively).

After adjustment for demographic, anthropometric/malnutrition (HAZ values), infection and environmental covariates, prevalence of low-severity anaemia was positively and significantly associated with *A. lumbricoides* and *T. trichiura* (OR = 1.18 and OR = 1.32, *p* = 0.04 and 0.02, respectively). *A. lumbricoides* (OR = 1.36, *p* = 0.01)*, T. trichiura* (OR = 1.43, *p* = 0.01), malaria (OR = 1.17, *p* = 0.00), NDVI (OR = 1.36, *p* = 0.01) and SES (OR = 1.36, *p* = 0.00) were all found to be positively and significantly associated with the prevalence of moderate/high-severity anaemia (Table 3).

### 3.3. Spatial Dependence in Prevalence of Anaemia and of Anaemia Severity Classes

Semivariograms for observed prevalence of anaemia demonstrated a trend in spatial dependency for all years except 2008 (Supplementary Materials, Figure S1). After accounting for all covariates, residual semivariograms of the prevalence of anaemia showed trends in spatial dependency for all years except 2011. Residual semivariograms for the prevalence of low-severity anaemia showed that clustering was evident in 2008 and 2010, whilst trends in spatial dependency were evident in 2009 and 2011 (Supplementary Materials, Figure S2). Residual semivariograms for the prevalence of moderate/high-severity anaemia showed that clustering was only evident in 2008, whilst trends in spatial dependency were evident from 2009 to 2011 (Supplementary Materials, Figure S3 and Table S2).

### 3.4. Spatial Risk Prediction

#### 3.4.1. Predicted Prevalence of Anaemia

Our analysis shows that for all years, boys had a higher prevalence of anaemia than girls (Table 4). The results of the prevalence of anaemia model indicate the presence of small clusters of anaemia prevalence throughout Burundi (*Φ* = 220.3, clusters ~1.52 km). 

**Figure 1:**
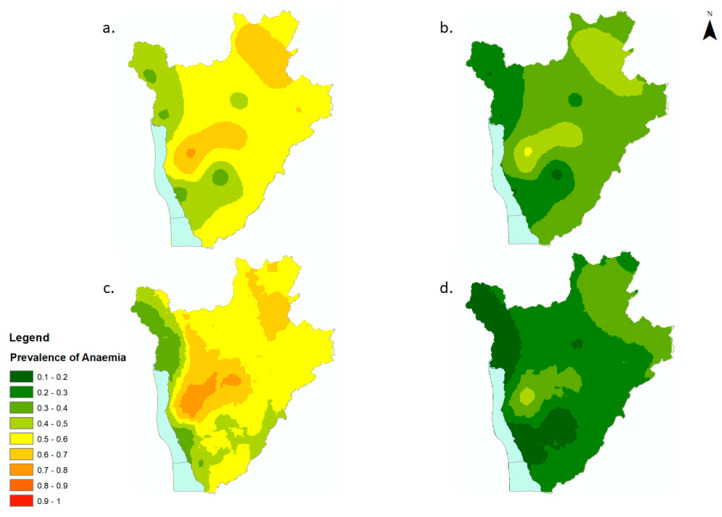
Predicted prevalence maps of anaemia per year for school-aged children (5–15 yrs) in Burundi, 2008–2011. (**a**) 2008, (**b**) 2009, (**c**) 2010, (**d**) 2011.

Variation in prevalence of anaemia was evident throughout the years and all over the country. High prevalence of anaemia (>50%) appeared to be concentrated within the central, northern and eastern regions of the country in 2008 and 2010 (Figure 1a,c). These areas maintained a moderate prevalence of anaemia (20–50%) in 2009 and 2011 (Figure 1b,d). A significant decrease in the prevalence of anaemia, from 40–50% in 2009 to 10–20% in 2011, was predicted in areas to the west of the country (Figure 1a,d). Maps for all years exhibited very low to low levels of uncertainty (Supplementary Materials, Figure S5).

**Figure 2:**
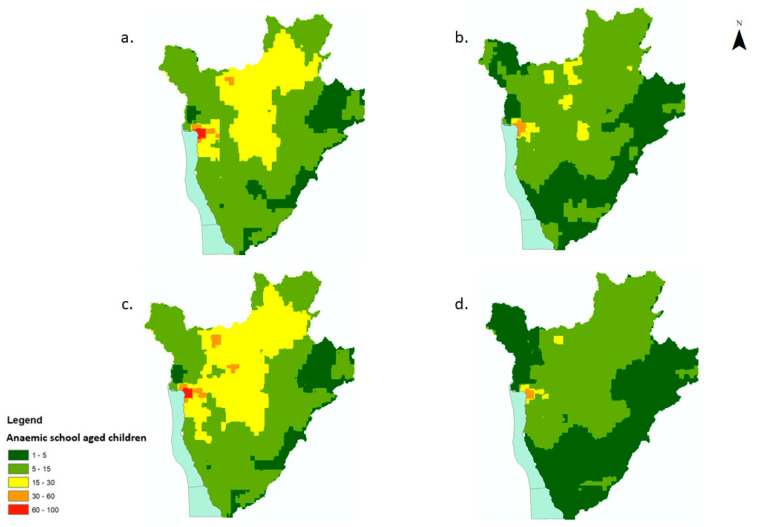
Estimated number of anaemic school-aged children (5–15 yrs) in Burundi, 2008–2011, per 550 m^2^ pixel. (**a**) 2008, (**b**) 2009, (**c**) 2010, (**d**) 2011.

Areas of high prevalence of anaemia corresponded to high numbers (>15 children with anaemia per 500 m^2^ pixel) of affected SAC (Figure 2a–d). The numbers of anaemic children per district and per year in Burundi are provided in Supplementary Materials, Table S3. The overall number of anaemic children decreased between 2008 and 2011 by 48%, from 443,657 to 232,304, with a resurgence in the number of anaemic children during the MDA disruption in 2010 (to 480,605, an increase of 8% from 2008, Figure 2a–d).

#### 3.4.2. Predicted Prevalence of Anaemia Severity Classes

Predicted prevalence of low- and moderate/high-severity anaemia was higher in SAC boys than in girls from 2008 to 2011. For 2008, the predicted prevalence of low-severity anaemia was 15% (boys) and 14% (girls), and that of moderate/high severity was 8% (boys) and 7% (girls). For 2011, the corresponding values for predicted low-severity anaemia were 8% (boys) and 7% girls, and for moderate/high severity, 3% (boys) and 2% (girls). These differences were statistically significant (*p* < 0.01) after adjusting for all covariates and including STH status.

The predicted prevalence of low-severity anaemia demonstrated smaller clusters (*Φ* = 14.47; 23.1 km) across the country, whilst prevalence of moderate/high-severity anaemia exhibited larger clusters across the country (*Φ* = 1.4, 238.5 km). 

**Figure 3:**
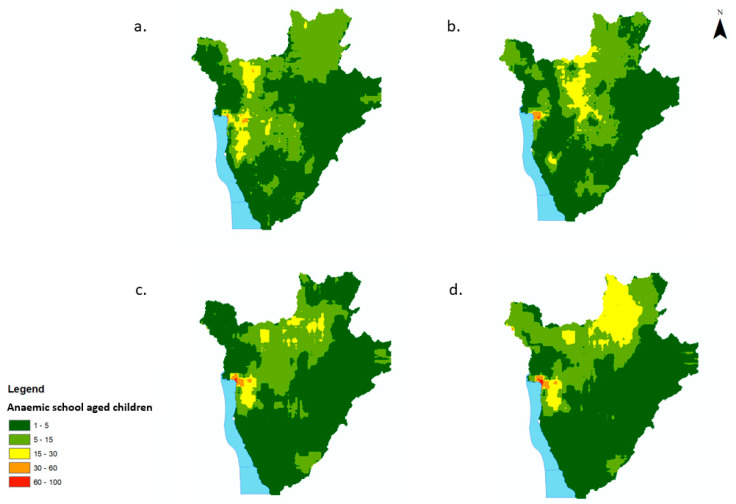
Predicted prevalence maps of low-severity anaemia per year for school-aged children (5–15 yrs) in Burundi, 2008–2011. (**a**) 2008, (**b**) 2009, (**c**) 2010, (**d**) 2011.

**Figure 4:**
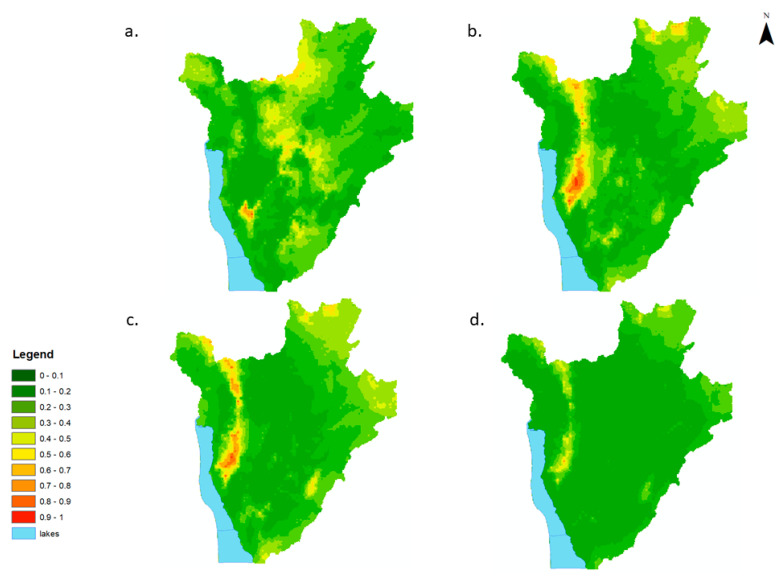
Predicted prevalence maps of moderate/high-severity anaemia per year for school-aged children (5–15 yrs) in Burundi, 2008–2011. (**a**) 2008, (**b**) 2009, (**c**) 2010, (**d**) 2011.

The predicted prevalence of low-severity anaemia appeared high (>70%) along the western region of the country in 2008, where it gradually receded until it remained concentrated in the central west region of the country (south east of the capital Bujumbura). Along the central north and northeast of the country, the predicted prevalence of low-severity anaemia increased from between 30–40% (2008) to >80% (2011). The predicted prevalence of low-severity anaemia appeared to decrease, from 40–50% (2008) to <20% in 2011 in the south and east of the country (Figure 3a–d). Areas with predicted prevalence of moderate/high-severity anaemia severity appeared to be concentrated in the western regions of Burundi between 2008 and 2011, with pockets of 40–50% predicted prevalence of moderate/high-severity anaemia in the central and northern regions in 2008. Areas with predicted prevalence of moderate/high-severity anaemia decreased in size throughout the country between 2008 and 2011 but remained at values between 30–40% in the western region of the country in 2011 (Figure 4a–d).

### 3.5. Estimates of the Number of Anaemic Children and Their Classification

Overall, the predicted number of children with either low- or moderate/high-severity anaemia decreased between 2008 and 2011, with a 1.3% reduction, from 219,628 to 216,813 for children with low-severity anaemia, and with a 60% reduction, from 175,066 to 70,453 for children with moderate/high-severity anaemia (Supplementary Materials, Table S4).

**Figure 5:**
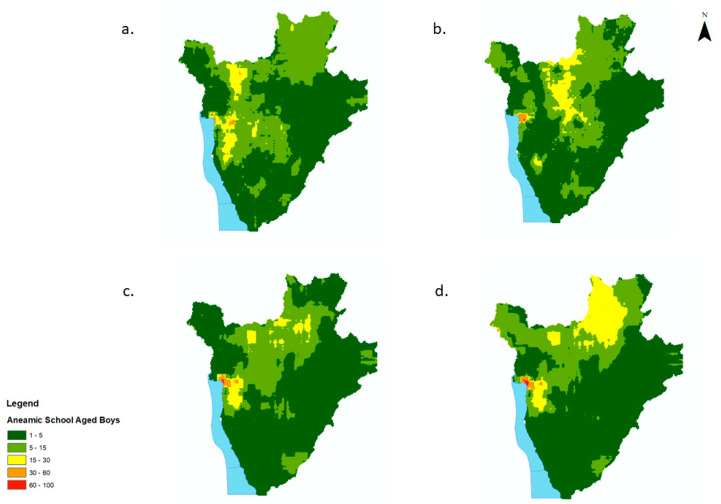
Estimated number of school-aged children (5–15 yrs) with low-severity anaemia in Burundi, 2008–2011, per 550 m^2^. (**a**) 2008, (**b**) 2009, (**c**) 2010, (**d**) 2011.

**Figure 6:**
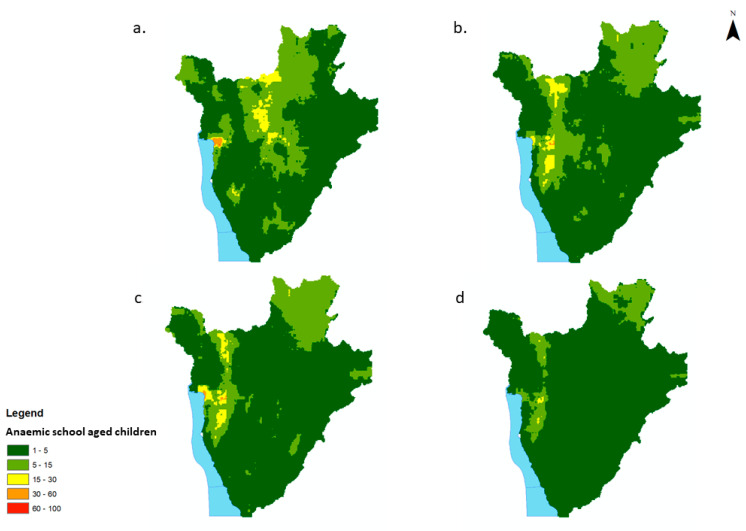
Estimated number of school-aged children (5–15 yrs) with moderate/high-severity anaemia in Burundi, 2008–2011, per 550 m^2^. (**a**) 2008, (**b**) 2009, (**c**) 2010, (**d**) 2011.

The number of children with low-severity anaemia was highest in and around the capital district, Bujumbura, for all years, with on average between 60–100 SAC children per pixel (550 m^2^) predicted to have low-severity anaemia (Figure 5a–d). The northern districts of Burundi experienced an increase in the number of children with low-severity anaemia in 2011 compared to 2008. Overall, there was a decrease in the number of children with moderate/high-severity anaemia from 2008 to 2011 (Figure 6a–d). The greatest burden was, again, predicted to be in and around the capital district of Bujumbura, with on average >50 children per pixel predicted to have moderate/severe anaemia.

Uncertainty around the predicted prevalence of low-severity anaemia was low (<5%). Predictive maps of the prevalence of moderate/high-severity anaemia also had low associated uncertainty across the years (Supplementary Materials, Figures S5 and S6). 

### 3.6. Model Validation

The models for prevalence of anaemia had a mean predictive error for all years ranging between −0.005 and 0.027, except for those in 2010, which had a mean predictive error of 0.18. The mean predictive error for the prevalence of anaemia severity models, for all years, ranged from 0.05 to 0.08 for low-severity anaemia, and from −0.009 to 0.12 for moderate/high-severity anaemia (Supplementary Materials, Table S5).

## 4. Discussion

In this study, we used a large longitudinal database from a 5-year STH MDA programme in Burundi to investigate spatiotemporal changes in prevalence of anaemia and of anaemia severity, and to explore the relationships between these and STH, while controlling for malaria endemicity, malnutrition, SES and environmental conditions. Overall, our results suggest substantial spatiotemporal variation in anaemia prevalence and severity over the 5-year MDA programme in Burundi. After adjusting for confounders and other factors contributing to anaemia, the results suggest that ascariasis, trichuriasis, malaria and NDVI are significantly influential on the prevalence and intensity of anaemia in Burundi. Our models predicted that areas with the highest burden of disease, and thus requiring the most attention, include areas around Bujumbura, the capital of Burundi and the central and northern districts of the country. Furthermore, children with *A. lumbricoides* were almost twice as likely to be anaemic than children with *T. trichiura.*

STH has 2 know causal pathways for co-morbidity in infected children (1) it causes malnutrition evidenced by stunting and wasting [21] and (2) directly causes anaemia [19]. Anaemia reduction, in both prevalence and severity, was evident countrywide from 2008 until 2010, after which there was an increase in anaemia prevalence and severity in 2010, followed by another decrease in prevalence and severity in 2011. Civil unrest in Burundi in 2010 led to all programmes in extension sites being halted due to security issues, and no treatment or data collection were possible in 19 out of 31 schools. Moreover, due to civil unrest, a substantial resurgence in the prevalence of anaemia and moderate/high-severity anaemia was observed in the same year, similar to baseline rates, highlighting the fragility of MDA-only programmes for STH morbidity control. This indicates that the role of MDA in reducing STH-related co-morbidities, and anaemia in particular, relies heavily on continuous treatment. Thus, the risk of developing anaemia remained high for SAC, especially boys, across the years. Interestingly, the civil unrest mainly involved regions near the capital on the central western border of the country. Patterns of migration suggest that people fleeing the civil unrest from urban areas migrated east towards the centre of the country [40,41], which is also the region with the greatest rise in anaemia prevalence in 2010 and 2011. This suggests that if human migration movements are not considered during health interventions, especially in places prone to instability, prevalence is likely to spike back to near-baseline levels in the affected areas, essentially reversing the impact of a long-running MDA programme within the course of a year.

Examining the relationship between anaemia and STH, after adjusting for malaria and other confounders and stratifying the prevalence of anaemia to reflect the prevalence of low- and moderate/high-severity anaemia yielded programmatically relevant results. We found that NDVI (negatively) was significantly associated with the prevalence of anaemia, whilst malaria and SES were both positively and significantly associated with the prevalence of moderate/high severity anaemia. This finding emphasises and supports current literature on the role of scarce vegetation coverage and the presence of malaria in increasing the prevalence of anaemia [42]. Low NDVI represents low vegetation coverage, however it is unknown from NDVI measures if this represents no-farming populations or farming areas experiencing crop failure and as such the exact causal pathway in which NDVI influences anaemia is largely unclear [42]. However, the positive association with SES could be due to SES data not being available per child and only being available at the school location via a proxy variable from the DHS [43]. This meant that our statistical models associated anaemia with a relatively higher SES (i.e., the school) area rather than the child’s home SES.

After adjusting for other covariates, infection by *A. lumbricoides* was found to be significantly associated with the prevalence of anaemia and the prevalence of both low- and moderate/high-severity anaemia. The biological ability of *A. lumbricoides* to produce c.200,000 eggs/day [44], causing inflammation in the intestinal tract, may result in increased prevalence of anaemia and explain the trend evident in our models. In fact, *A. lumbricoides* eggs in tissue incite granulomatous inflammation, and larvae migrating through the host’s lungs stimulate an inflammatory infiltrate with abundant eosinophilic leukocytes. Our results also show that *T. trichiura* infections were significantly associated with the prevalence of anaemia and the prevalence of low-severity anaemia and moderate-high severity anaemia. *T. trichiura* infections often co-occur together with *A. lumbricoides* in Burundi [24,45]. *T. trichiura* female worms produce approximately 20,000 eggs per day [45], and their anterior end is embedded in epithelial cells within the intestinal mucosa, causing substantial inflammation at the site of attachment. Contrary to common understanding and knowledge of the relationship between anaemia and STH, and thus somewhat surprisingly, hookworm was not significantly associated with any of the anaemia prevalence indicators used. In our previous studies, we had reported that, in Burundi, hookworm infection was classed as belonging to the low- and moderate-intensity classes according to the WHO [24]. The lack of association with anaemia prevalence found here could be partly explained by hookworm infections being more prevalent in adults than children [46], and as such we would not expect to see a large effect of hookworm infection on anaemia in SAC. Furthermore, whilst hookworm was moderately prevalent in Burundi, we had also reported that hookworm co-infections made up less than 10% of co-infection patterns [24]. 

Our approach represents an important advancement in the way we evaluate the effectiveness of STH MDA programmes aimed at morbidity reduction by adding a spatiotemporal component to describe changes in the number of children at risk of anaemia both geographically and temporally. Using this approach, our results suggest significant spatial heterogeneity in anaemia prevalence and severity after controlling for covariates. Additionally, despite the 5-year MDA programme and evident reduction of anaemia over time, pockets of high prevalence of anaemia and moderate/high-severity anaemia remained in the country south-east of Bujumbura and in the northern and central northern districts of the country. Our spatiotemporal predictive maps of anaemia prevalence and severity allow the identification of communities in need of public health interventions, both STH MDA and anaemia specific, which can be lifesaving in the cases of severe anaemia. Using such maps in the context of STH MDA programmes can assist monitoring and evaluation as well as disease surveillance activities. The opportune availability of such maps would have benefited the Burundi MDA programme, by alerting that despite ongoing MDA, the prevalence of anaemia remained high, and increased over the years in parts of Burundi where MDA was disrupted. 

Comparing the results reported in this paper to those of the predicted number of children infected with each nematode species responsible for STH reported previously for Burundi [25], it is reassuring to see that if the numbers of children predicted to be infected with each parasitic infection were low, they generally corresponded with low numbers of children with anaemia in both severity classes. Areas with the highest density of low- and moderate/severe anaemia (>60 children per pixel) corresponded to areas where both the number of children with *A. lumbricoides* and *T. trichiura* were highest (>80 children per pixel), and these areas were predominantly in and around Bujumbura and northern districts of Burundi.

Furthermore, our results indicate that prevalence of anaemia was higher in SAC boys than girls for all years of this study. While again, somewhat unexpected [17], this finding may reflect the higher risk of STH acquisition by boys as a result of activities related to being outdoors at an earlier age and more often than girls, or helping adults in farming, fishing and/or agricultural work [47]. However, since the models adjusted for STH species, it is also likely that differences in diet [22], glucose-6-phosphate dehydrogenase (G6PD deficiency—a genetic disorder which affects males more often) [48] or HIV [49] can play key roles in anaemia in boys. Due to limitations in available data, the aforementioned could not be accounted for in the models. This finding has potentially important programmatic implications since the majority of anaemia interventions are geared towards women of childbearing age [50,51] and requires further investigation. 

## 5. Study Limitations

These are categorised as those due to consideration of other infections, diagnostic accuracy (of both STH and anaemia) and sample size limitations. 

*Other infections*: A degree of caution is required when interpreting the anaemia prevalence models presented here as not all conditions and infections potentially associated with anaemia were included in the models, e.g., HIV infection. Due to the stigmatisation accompanying HIV diagnosis and barriers to treatment access, data and maps of HIV prevalence do not exist in a high enough resolution to have allowed for incorporation of this information into the models. Malaria data were obtained from MAP (https://map.ox.ac.uk/, accessed on 15 September 2017). These maps contained large areas of missing data and, hence, an interpolation method was used to predict the missing areas using nearest neighbour cell prevalence values as a proxy for the missing cells. This probably introduced error into the prediction models that used malaria prevalence as one of the covariates. The areas for which malaria prevalence information was interpolated overlapped with areas of low to moderate uncertainty in the anaemia maps across all years.

*Diagnostic accuracy (of STH and anaemia)*: To account for diagnostic problems with hookworm larvae deteriorating rapidly if slides are not read promptly, two slides per sample were taken for Kato-Katz testing, with one slide tested within 30 min but the other slide set to rest, which could have affected the sensitivity of diagnosis for hookworm infection [24,25]. The use of a qualitative test to collect Hb levels in 2011 introduced a source of error into the quantification of Hb levels. Whilst a correction was applied to the data collected in 2011, the more subjective nature of qualitative results may have imposed a limit to the ability of these corrections to provide accurate results. 

*Sample size*: The sample size available for analysis in 2010 was considerably smaller than those for other years of the study due to political instability in Burundi, which led to no treatment received in all extension sites, and thus no data collected from those sites.

*Adjusting population density map:* Population maps used in our models have been adjusted using general annual growth rates and as such they are subject to accuracy issues as actual annual growth rates may have not been homogenous across the entire nation. Moreover, data were predicted to 550 m × 550 m resolution. The clear limitation of this is that the population density will vary within 5 km × 5 km and by predicting the prevalence at a higher resolution, the number of infected people will carry a margin of error. Urban-rural ratios were not applied to the population density models to prevent under estimation of number of anaemic children. However, by not accounting for urban-rural ratios produces bias towards high population centres (urban areas) and introduces a margin of error.

*Unaccounted for uncertainty:* SES data from DHS and malaria data were collected from sources that were not designed to provide accurate focal point estimates and as a such introduce a degree of uncertainty in prediction models. Adjusting for malnutrition would have reduced the observed effect of STH on anaemia, however due to limitations of available data, no HAZ or WAZ scores for children over the age of 5 were available.

## 6. Conclusions

We have made maximum use of monitoring and evaluation data, collected by the SCI-assisted Ministry of Health STH MDA programme in Burundi between 2008 and 2011, by combining statistical models for the relationship between anaemia and STH after adjusting for a number of covariates with spatiotemporal analysis of the predicted prevalence of anaemia and anaemia severity classes. The models indicated, perhaps surprisingly, that ascariasis and trichuriasis were positively and significantly associated with anaemia whereas hookworm infection was not, and that SAC boys were at higher risk of developing anaemia than girls. Despite substantial efforts to reduce morbidity associated with STH by the implementation of a national MDA programme, the risk of anaemia in SAC boys remained high and in fact increased in certain parts of the country, likely due to migration patterns during political unrest. Thus, it is recommended that MDA programmes aiming at morbidity reduction incorporate specific anaemia interventions, such as iron fortification of food or iron supplementation, alongside anthelmintic interventions, and flexibly respond to the changing needs of the population at risk, particularly after civil unrest has disrupted the continuity of the MDA programme and elicited large-scale movement of targeted populations.

## Figures and Tables

**Table 1:** Prevalence of soil-transmitted helminthiases in school-aged children classified according to their anaemia status in Burundi, 2008–2011.

Parasite	Not Anaemic		Anaemic	
	Prevalence	95% CI	Prevalence	95% CI
*Ascaris lumbricoides*	11.0%	(6.7, 15.3)	17.0%	(11.2, 22.8)
*Trichuris trichiura*	4.4%	(2.8, 6.1)	6.3%	(3.2, 9.4)
Hookworm	7.5%	(5.9, 9.4)	8.8%	(6.1, 11.6)

**Table 2:** Prevalence of soil-transmitted helminthiases in school-aged children classified according to their anaemia severity in Burundi, 2008–2011.

Anaemia Severity	*Ascaris lumbricoides*		*Trichuris trichiura*		Hookworm	
	Prevalence	95% CI	Prevalence	95% CI	Prevalence	95% CI
None	11.0%	(6.7, 15.3)	4.4%	(2.8, 6.1)	7.5%	(5.5, 9.4)
Low	15.1%	(9.8, 20.4)	6.3%	(2.9, 9.7)	9.1%	(6.4, 11.8)
Moderate	19.2%	(12.7, 25.8)	5.1%	(2.7, 7.5)	8.5%	(5.1, 11.8)
High	23.0%	(3.4, 42.7)	15.1%	(4.6, 25.6)	8.1%	(3.4, 12.8)

**Table 3:** Results of the final non-spatial statistical models for factors associated with the prevalence of anaemia and of anaemia severity in Burundi, 2008–2011.

	Prevalence of Anaemia		Prevalence of Low Severity Anaemia		Prevalence of Moderate/High Severity Anaemia	
Variable	Odds-Ratio	*p*-Value	Odds-Ratio	*p*-Value	Odds-Ratio	*p*-Value
Gender—male	1.27	0.00	1.24	0.00	1.14	0.00
*A. lumbricoides*	1.56	0.00	1.18	0.04	1.36	0.01
*T. trichiura*	1.39	0.00	1.32	0.02	1.43	0.01
Hookworm	1.09	0.40	1.11	0.20	1.08	0.31
Malaria	0.96	0.74	0.93	0.32	1.17	0.00
LST	1.21	0.41	1.31	0.24	0.75	0.48
DPWB	1.16	0.05	1.01	0.97	0.73	0.22
NDVI	0.90	0.00	1.12	0.25	1.36	0.01
Precipitation	0.89	0.11	0.93	0.50	0.76	0.17
Elevation	1.63	0.03	1.68	0.07	1.23	0.66
SES	0.99	0.77	1.09	0.29	1.36	0.00

**Table 4:** Prevalence of anaemia for school-aged children in Burundi.

	Prevalence of Anaemia (Boys)	(95% CI)	Prevalence of Anaemia (Girls)	(95% CI)	Total
2007	38%	(0.33, 0.42)	33%	(0.28, 0.37)	35%
2008	49%	(0.45, 0.54)	45%	(0.40, 0.49)	47%
2009	29%	(0.25, 0.33)	27%	(0.23, 0.31)	28%
2010	40%	(0.35, 0.44)	35%	(0.31, 0.39)	37%
2011	23%	(0.19, 0.26)	18%	(0.14, 0.21)	20%
Overall years	33%	(0.29, 0.38)	29%	(0.25, 0.33)	31%

## Data Availability

The data presented in this study are available on request from the corresponding author. The data are not publicly available due to legal and privacy restrictions.

## Supplementary Materials

The following supporting information can be downloaded at: https://www.mdpi.com/article/10.3390/tropicalmed7100307/s1, Figure S1: Semivariograms for the prevalence of anaemia in Burundi, 2008–2011; Figure S2: Semivariograms for the prevalence of low-severity anaemia in Burundi, 2008–2011; Figure S3: Semivariograms for the prevalence of moderate/high-severity anaemia in Burundi, 2008–2011; Figure S4: Standard deviation maps of anaemia prevalence per year in Burundi, 2008–2011; Figure S5: Standard deviation maps for the prevalence of low-severity anaemia per year in Burundi, 2008–2011; Figure S6: Standard deviation maps for the prevalence of moderate/high-severity anaemia per year in Burundi, 2008–2011; Table S1: Anaemia severity thresholds (blood haemoglobin concentration) [52]; Table S2: Results of semivariogram parameters for the prevalence of anaemia, and the prevalence of anaemia severity in Burundi per year, 2008–2011; Table S3: Estimated number of anaemic school-aged children per district in Burundi, 2008–2011; Table S4: Estimated number of school-aged children with low- and moderate/high-severity anaemia per district in Burundi, 2008–2011; Table S5: Values of the validation metrics used for the predictive maps of the prevalence of anaemia and of anaemia severity clas- ses in school-aged children in Burundi, 2008–2011.

## Abbreviations

ASTER, Advanced Spaceborne Thermal Emission and Reflection Radiometer; CIESIN, Center for International Earth Science Information Network; DHS, Demographic Health Survey; DPWB, Distance to perennial water body; GDEM, Global Digital Elevation Map; GIS, Geographical Information System; GPS, Global Positioning System; GRUMP, Global Rural Urban Mapping Project; HAZ, Height-for-age Z-scores; Hb, haemoglobin; IDW, inverse distance weighting; LST, Land Surface Temperature; MAP, Malaria Atlas Project; MBG, model-based geostatistics; MDA, mass drug administration; NDVI, Normalized Differential Vegetation Index; OR, odds ratio; SAC, school-aged children; SCI, Schistosomiasis Control Initiative; SES, socioeconomic status; STH, soil-transmitted helminthiases; WHO, World Health Organization; 95% CI, ninety-five percent credible interval.
