# Supplementary material for: Impact of a 5-Year Mass Drug Administration Programme for Soil-Transmitted Helminthiases on the Spatial Distribution of Childhood Anaemia in Burundi from 2007 to 2011
Source: Trop Med Infect Dis. 2022 Oct 17;7(10):307. doi: 10.3390/tropicalmed7100307 (PMC9611614; doi:10.3390/tropicalmed7100307)
Supplement: Supplementary file 1 [file tropicalmed-07-00307-s001.zip › tropicalmed-1939829-supplementary.pdf]

**Annex**

**Tables:**

*Table S1 : Anaemia severity thresholds (blood haemoglobin concentration).*

| Population age groups and status | Non-anaemic (g/l) | Low severity (g/l) | Moderate severity (g/l) | High severity (g/l) |
|---|---|---|---|---|
| 5–11yrs | ≥115 | 110–114 | 80–109 | <80 |
| 12–14yrs | ≥120 | 110–119 | 80–109 | <80 |
| Non-pregnant women aged ≥15yrs | ≥120 | 110–119 | 80–109 | <80 |
| Pregnant women aged ≥15yrs | ≥110 | 100–109 | 70–99 | <70 |
| Men aged ≥15yrs | ≥130 | 110–129 | 80–109 | <80 |

Classification is according to the World Health Organization guidelines [33].

*Table S2: Results of semivariogram parameters for the prevalence of anaemia, and the prevalence of anaemia severity in Burundi per year, 2008–2011.*

| Year | Raw anaemia | Anaemia residuals | Low severity | Moderate/high severity |
|---|---|---|---|---|
| **2008** | | | | |
| Spatial autocorrelation | None | Trended | Clustered | Clustered |
| Nugget | 0.0712379 | 0.00469545 | 0 | 0 |
| Sill | 0 | 0.008014904 | 0.008307482 | 0.01295277 |
| Range | 1.180104 | 0.9034204 | 0.5694854 | 0.3049023 |
| **2009** | | | | |
| Spatial autocorrelation | Trended | Trended | Trended | Trended |
| Nugget | 0.015418361 | 0.001782568 | 0.001611758 | 0 |
| Sill | 0.002902369 | 0.010901082 | 0.004095228 | 0.006998316 |
| Range | 0.9312157 | 1.198152 | 1.798028 | 0.8986704 |
| **2010** | | | | |
| Spatial autocorrelation | Trended | Trended | Clustered | Trended |
| Nugget | 0 | 0.01044786 | 0 | 0.01955982 |
| Sill | 0.1884992 | 0.18751196 | 0.02849971 | 0.07767505 |
| Range | 0.8221483 | 0.9977894 | 0.2618964 | 1.135447 |
| **2011** | | | | |
| Spatial autocorrelation | None | Trended | Trended | Trended |
| Nugget | 0.02082441 | 0 | 0 | 0 |

| | | | | |
|---|---|---|---|---|
| Sill | 0 | 0.009436121 | 0.005866115 | 0.00221214 |
| Range | 0.96954 | 1.198615 | 1.797464 | 0.8987203 |

*Table S3: Estimated number of anaemic school-aged children per district in Burundi, 2008–2011.*

| | Number of anaemic school-aged children | | | |
|---|---|---|---|---|
| **District** | **2008** | **2009** | **2010** | **2011** |
| Bubanza | 8,504 | 5,051 | 8,922 | 4,005 |
| Buhiga | 14,101 | 9,478 | 15,893 | 7,734 |
| Bururi | 5,463 | 3,239 | 6,235 | 2,408 |
| Busoni | 9,064 | 6,152 | 9,492 | 5,215 |
| Butezi | 8,963 | 6,181 | 9,520 | 4,573 |
| Buye | 10,549 | 7,054 | 11,072 | 5,487 |
| Cankuzo | 6,562 | 4,584 | 6,786 | 3,764 |
| Cibitoke | 8,885 | 5,056 | 8,284 | 3,833 |
| Fota | 9,287 | 6,362 | 11,182 | 5,409 |
| Gahombo | 9,068 | 5,892 | 9,977 | 4,521 |
| Gashoho | 8,902 | 6,350 | 9,335 | 5,235 |
| Gihofi | 7,927 | 5,265 | 8,203 | 3,911 |
| Gitega | 14,474 | 10,046 | 16,612 | 7,811 |
| Giteranyi | 12,955 | 9,016 | 13,510 | 7,492 |
| Isale | 15,231 | 9,206 | 15,374 | 7,065 |
| Kabezi | 9,441 | 6,101 | 10,000 | 5,128 |
| Kayanza | 13,638 | 8,346 | 15,979 | 7,032 |
| Kibumbu | 9,603 | 6,707 | 11,582 | 5,509 |
| Kibuye | 13,285 | 8,900 | 15,350 | 6,821 |
| Kiganda | 8,704 | 5,726 | 10,219 | 4,523 |
| Kinyinya | 9,294 | 6,674 | 9,254 | 5,084 |
| Kiremba | 15,455 | 10,604 | 16,186 | 8,545 |
| Kirundo | 10,846 | 7,411 | 11,095 | 6,089 |
| Mabayi | 10,736 | 6,145 | 11,247 | 4,767 |
| Makamba | 12,279 | 8,112 | 12,793 | 6,273 |
| Matana | 11,268 | 7,372 | 13,948 | 6,075 |
| Mpanda | 7,364 | 4,407 | 7,405 | 3,331 |
| Mukenke | 7,859 | 5,513 | 8,079 | 4,637 |
| Muramvya | 7,411 | 4,642 | 8,963 | 3,889 |
| Murore | 6,219 | 4,450 | 6,526 | 3,969 |
| Musema | 13,372 | 8,450 | 15,509 | 6,577 |
| Mutaho | 10,986 | 7,202 | 12,633 | 5,392 |
| Muyinga | 15,034 | 10,794 | 15,814 | 8,695 |
| Ngozi | 14,474 | 9,535 | 15,781 | 7,491 |
| Nyabikere | 12,174 | 7,982 | 13,903 | 6,059 |
| Nyanza-Lac | 9,212 | 5,890 | 9,157 | 4,622 |
| Rumonge | 11,755 | 7,489 | 12,096 | 5,730 |
| Rutana | 8,994 | 5,953 | 9,723 | 4,330 |

| | | | | |
|---|---|---|---|---|
| Ruyigi | 5,979 | 4,185 | 6,276 | 3,244 |
| Rwibaga | 8,121 | 5,473 | 9,599 | 5,022 |
| Ryansoro | 7,475 | 4,934 | 9,265 | 3,980 |
| Vumbi | 10,749 | 7,486 | 10,758 | 5,967 |
| ZONE-Centre | 2,115 | 1,233 | 1,964 | 890 |
| ZONE-Nord | 6,742 | 3,856 | 6,176 | 2,839 |
| ZONE-Sud | 3,135 | 1,865 | 2,927 | 1,335 |
| **Total** | **443,657** | **292,370** | **480,605** | **232,304** |

*Table S4: Estimated number of school-aged children with low- and moderate/high-severity anaemia per district in Burundi, 2008–2011.*

| | Low-severity anaemia | | | | Moderate/high-severity anaemia | | | |
|---|---|---|---|---|---|---|---|---|
| **District** | **2008** | **2009** | **2010** | **2011** | **2008** | **2009** | **2010** | **2011** |
| Bubanza | 4,915 | 2,815 | 2,485 | 3,984 | 2,082 | 3,639 | 4,323 | 2,177 |
| Buhiga | 4,882 | 5,774 | 8,525 | 10,009 | 4,308 | 3,359 | 2,993 | 1,156 |
| Bururi | 3,299 | 1,799 | 127 | 54 | 1,476 | 2,079 | 1,912 | 710 |
| Busoni | 6,402 | 3,891 | 2,483 | 8,516 | 3,529 | 5,695 | 6,421 | 4,578 |
| Butezi | 2,351 | 5,724 | 781 | 565 | 4,812 | 1,444 | 1,445 | 550 |
| Buye | 3,473 | 8,655 | 6,743 | 7,586 | 7,523 | 1,940 | 1,224 | 447 |
| Cankuzo | 3,348 | 2,246 | 1,573 | 1,106 | 1,725 | 2,836 | 3,029 | 1,335 |
| Cibitoke | 2,862 | 5,149 | 2,633 | 7,600 | 4,394 | 2,198 | 3,467 | 1,427 |
| Fota | 7,066 | 2,268 | 2,641 | 2,251 | 1,678 | 4,598 | 2,727 | 1,061 |
| Gahombo | 2,494 | 6,930 | 4,757 | 4,252 | 5,748 | 1,160 | 536 | 144 |
| Gashoho | 4,341 | 3,989 | 5,420 | 8,626 | 3,235 | 3,335 | 3,186 | 1,482 |
| Gihofi | 2,950 | 4,253 | 3,092 | 2,466 | 3,579 | 1,944 | 2,475 | 1,014 |
| Gitega | 6,170 | 7,951 | 2,117 | 1,429 | 6,112 | 2,959 | 912 | 239 |
| Giteranyi | 7,837 | 4,964 | 3,284 | 7,176 | 4,235 | 6,610 | 7,743 | 4,968 |
| Isale | 7,041 | 6,201 | 9,960 | 10,284 | 5,334 | 4,978 | 5,945 | 2,555 |
| Kabezi | 6,619 | 2,017 | 6,299 | 5,605 | 1,628 | 5,018 | 5,584 | 2,816 |
| Kayanza | 13,000 | 5,231 | 8,027 | 9,488 | 4,138 | 9,331 | 6,679 | 3,764 |
| Kibumbu | 5,419 | 4,248 | 2,581 | 1,647 | 3,201 | 2,803 | 967 | 236 |
| Kibuye | 6,406 | 5,984 | 3,546 | 2,840 | 4,776 | 3,258 | 1,997 | 645 |
| Kiganda | 3,974 | 5,682 | 4,183 | 3,218 | 4,651 | 2,358 | 1,205 | 436 |
| Kinyinya | 2,296 | 3,251 | 774 | 454 | 2,678 | 1,800 | 2,987 | 1,256 |
| Kiremba | 5,423 | 11,544 | 13,071 | 17,721 | 10,028 | 3,806 | 3,411 | 1,536 |
| Kirundo | 6,643 | 5,588 | 2,906 | 13,373 | 5,126 | 5,802 | 6,419 | 4,136 |
| Mabayi | 7,786 | 6,489 | 4,884 | 8,937 | 5,884 | 6,324 | 6,730 | 3,954 |
| Makamba | 6,139 | 6,174 | 6,726 | 5,112 | 5,322 | 4,220 | 4,426 | 2,016 |
| Matana | 8,174 | 2,978 | 1,474 | 1,649 | 2,188 | 5,202 | 2,739 | 978 |
| Mpanda | 3,002 | 3,709 | 2,151 | 3,101 | 2,988 | 2,147 | 2,640 | 1,070 |
| Mukenke | 4,362 | 3,348 | 2,864 | 7,144 | 2,934 | 3,662 | 4,476 | 2,835 |
| Muramvya | 6,255 | 2,211 | 3,756 | 3,326 | 1,704 | 4,363 | 3,494 | 1,674 |
| Murore | 4,126 | 1,803 | 3,785 | 2,924 | 1,464 | 3,691 | 4,376 | 2,480 |
| Musema | 6,025 | 9,104 | 6,640 | 5,686 | 7,393 | 3,433 | 1,921 | 695 |
| Mutaho | 2,397 | 8,761 | 3,400 | 2,556 | 6,988 | 1,199 | 580 | 151 |
| Muyinga | 7,558 | 5,065 | 2,859 | 3,389 | 3,759 | 6,108 | 6,111 | 2,726 |
| Ngozi | 4,267 | 8,708 | 8,189 | 8,517 | 7,156 | 1,999 | 1,185 | 348 |
| Nyabikere | 2,658 | 7,203 | 2,970 | 2,973 | 5,521 | 1,538 | 1,127 | 376 |
| Nyanza-Lac | 4,060 | 2,106 | 651 | 211 | 1,727 | 2,464 | 3,512 | 1,318 |
| Rumonge | 6,078 | 6,111 | 689 | 414 | 5,071 | 4,296 | 3,582 | 1,352 |
| Rutana | 4,866 | 3,124 | 2,361 | 1,916 | 2,604 | 2,777 | 3,097 | 1,317 |
| Ruyigi | 1,942 | 2,267 | 594 | 311 | 1,836 | 1,410 | 1,793 | 759 |

| | | | | | | | | |
|---|---|---|---|---|---|---|---|---|
| Rwibaga | 9,308 | 513 | 7,389 | 7,316 | 382 | 7,753 | 7,474 | 4,414 |
| Ryansoro | 4,089 | 3,478 | 2,149 | 1,498 | 2,709 | 2,160 | 1,030 | 336 |
| Vumbi | 4,199 | 7,730 | 5,563 | 13,072 | 7,039 | 3,341 | 3,684 | 1,939 |
| Zone-Centre | 555 | 1,070 | 1,014 | 1,111 | 954 | 373 | 586 | 186 |
| Zone-Nord | 1,805 | 2,847 | 3,855 | 4,579 | 2,596 | 1,167 | 2,059 | 629 |
| Zone-Sud | 765 | 1,043 | 782 | 820 | 848 | 511 | 813 | 231 |
| **Total** | **219,628** | **211,995** | **172,755** | **216,813** | **175,066** | **153,086** | **145,020** | **70,453** |

*Table S5: Values of the validation metrics used for the predictive maps of the prevalence of anaemia and of anaemia severity classes in school-aged children in Burundi, 2008–2011.*

| **Prevalence of anaemia** | **Mean prediction error** | **Absolute prediction error** |
|---|---|---|
| 2008 | -0.003 | 0.222 |
| 2009 | -0.005 | 0.112 |
| 2010 | -0.164 | 0.184 |
| 2011 | 0.027 | 0.104 |
| **Prevalence of low-severity anaemia** | | |
| 2008 | 0.063 | 0.232 |
| 2009 | 0.074 | 0.120 |
| 2010 | 0.086 | 0.146 |
| 2011 | 0.052 | 0.220 |
| **Prevalence of moderate/high-severity anaemia** | | |
| 2008 | 0.122 | 0.142 |
| 2009 | -0.009 | 0.107 |
| 2010 | 0.006 | 0.111 |
| 2011 | 0.021 | 0.046 |

**Figures:**

Semivariograms and maps for the standard deviation of the prevalence of anaemia and of anaemia severity in school-aged children in Burundi, 2008–2011, and tables with the predicted number of school-aged children with anaemia or in anaemia severity classes.

*Figure S1: Semivariograms for the prevalence of anaemia in Burundi, 2008–2011*

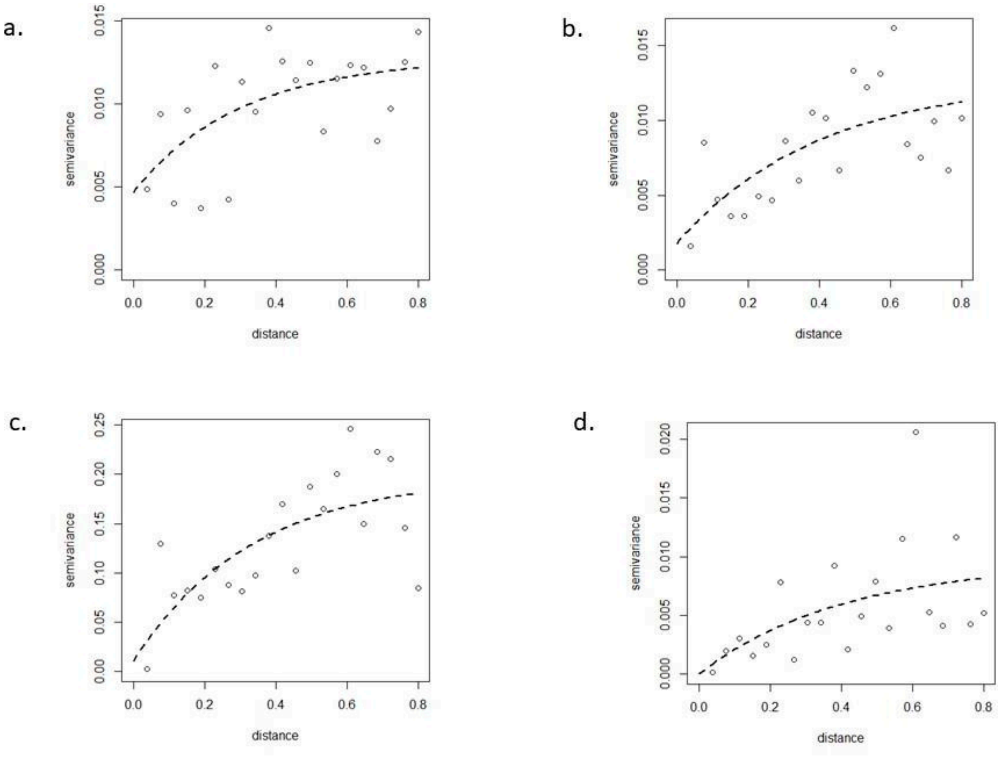


The y-axis represents the semivariance and the x-axis the distance (in decimal degrees). **(a)** 2008, **(b)** 2009, **(c)** 2010, **(d)** 2011.

*Figure S2:Semivariograms for the prevalence of low-severity anaemia in Burundi, 2008–2011.*

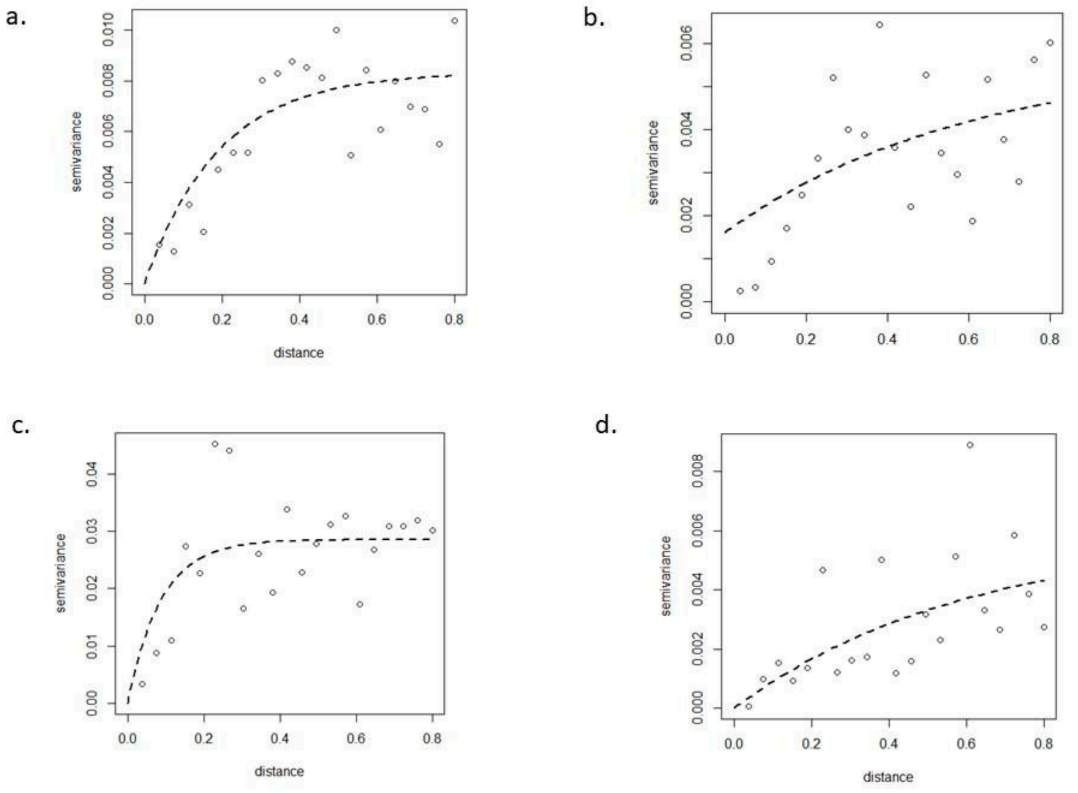


The y-axis represents the semivariance and the x-axis the distance (in decimal degrees). **(a)** 2008, **(b)** 2009, **(c)** 2010, **(d)** 2011.

*Figure S3: Semivariograms for the prevalence of moderate/high-severity anaemia in Burundi, 2008–2011.*

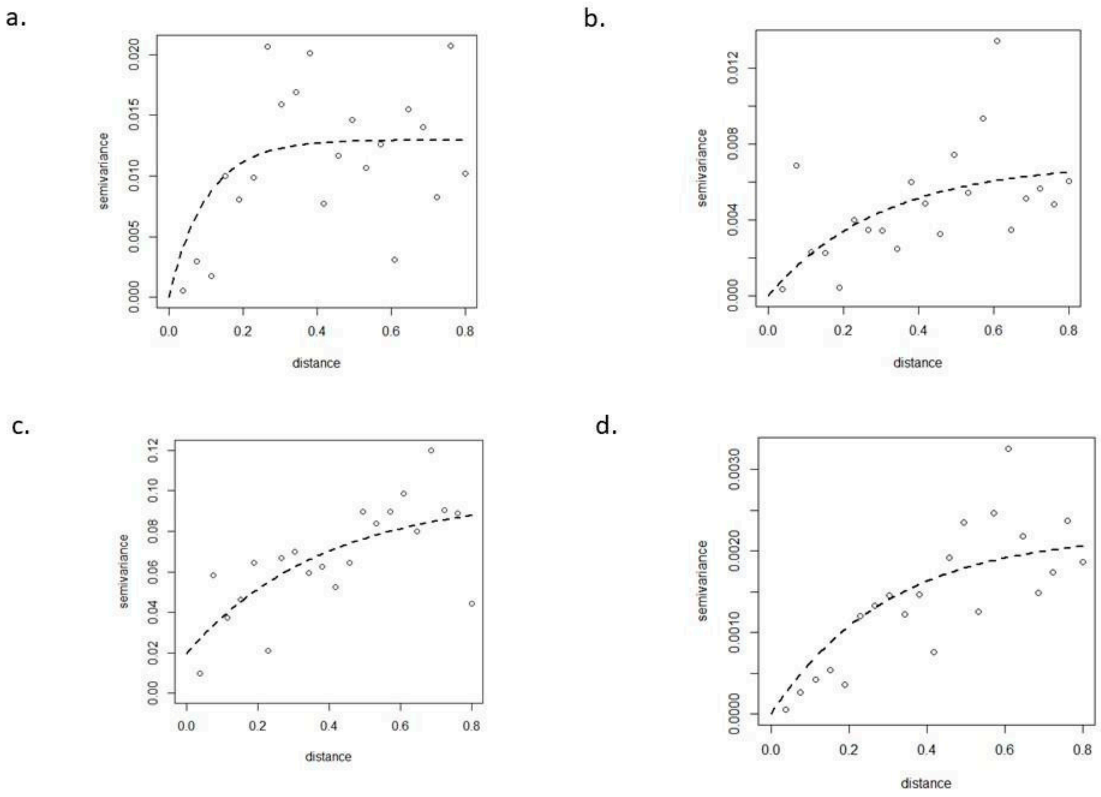


The y-axis represents the semivariance and the x-axis the distance (in decimal degrees). **(a)** 2008, **(b)** 2009, **(c)** 2010, **(d)** 2011.

*Figure S4: Standard deviation maps of anaemia prevalence per year in Burundi, 2008–2011.*

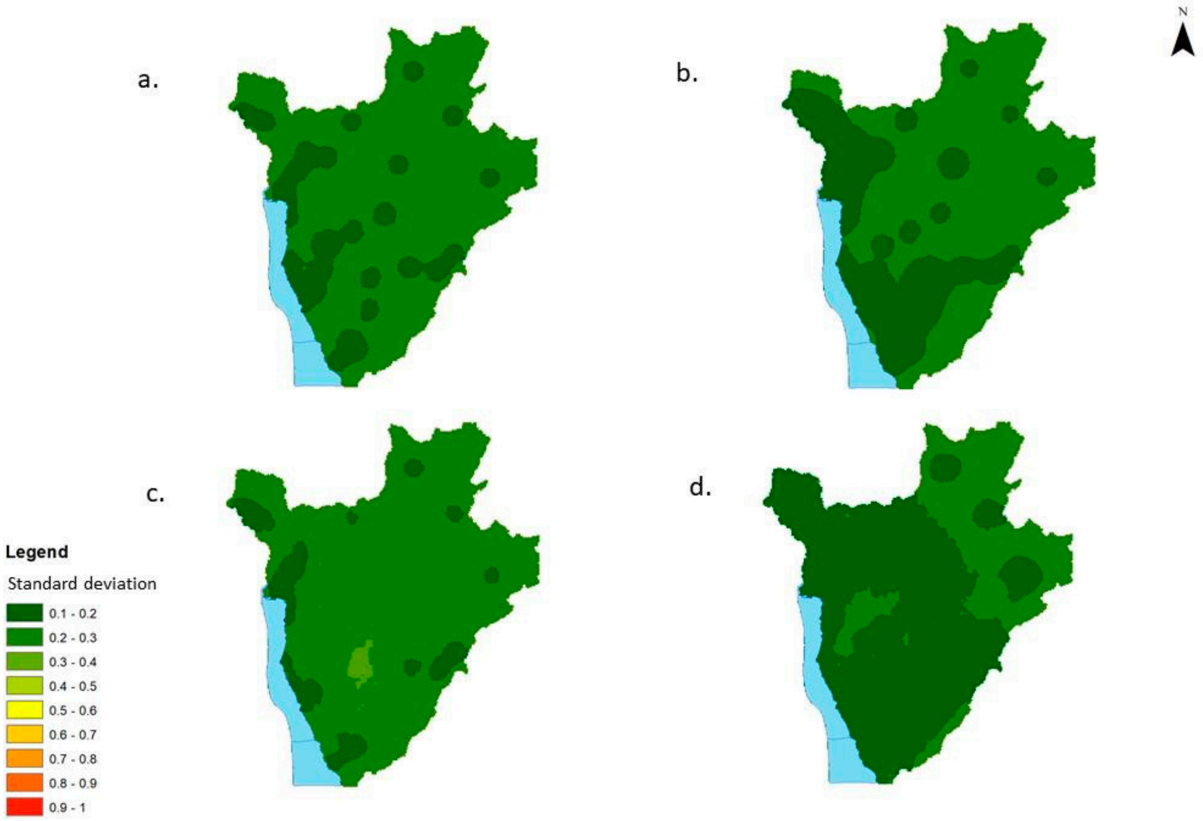


The intensity of the colour represents the magnitude of the standard deviation (see accompanying legend) for the proportion of school-aged children with anaemia (see Table 1 of main text for classification of anaemia according to the World Health Organization [1]). The pale blue colour depicts the location Lake Tanganyika. **(a)** 2008, **(b)** 2009, **(c)** 2010, **(d)** 2011.

*Figure S5: Standard deviation maps for the prevalence of low-severity anaemia per year in Burundi, 2008–2011.*

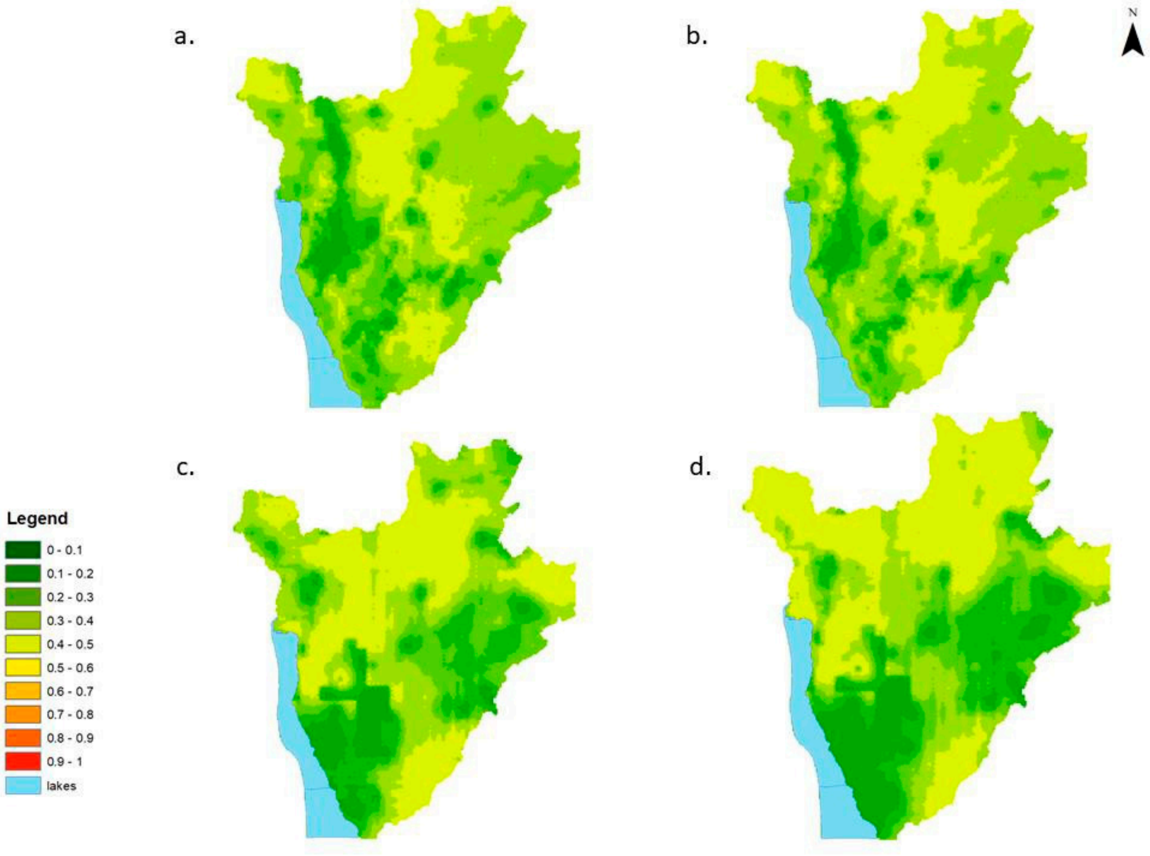


The intensity of the colour represents the magnitude of the standard deviation (see accompanying legend) for the proportion of school-aged children with low-severity anaemia (see Table 1 of main text for classification of anaemia and anaemia severity categories according to the World Health Organization [1]). The pale blue colour depicts the location of Lake Tanganyika. **(a)** 2008, **(b)** 2009, **(c)** 2010, **(d)** 2011.

*Figure S6: Standard deviation maps for the prevalence of moderate/high-severity anaemia per year in Burundi, 2008–2011.*

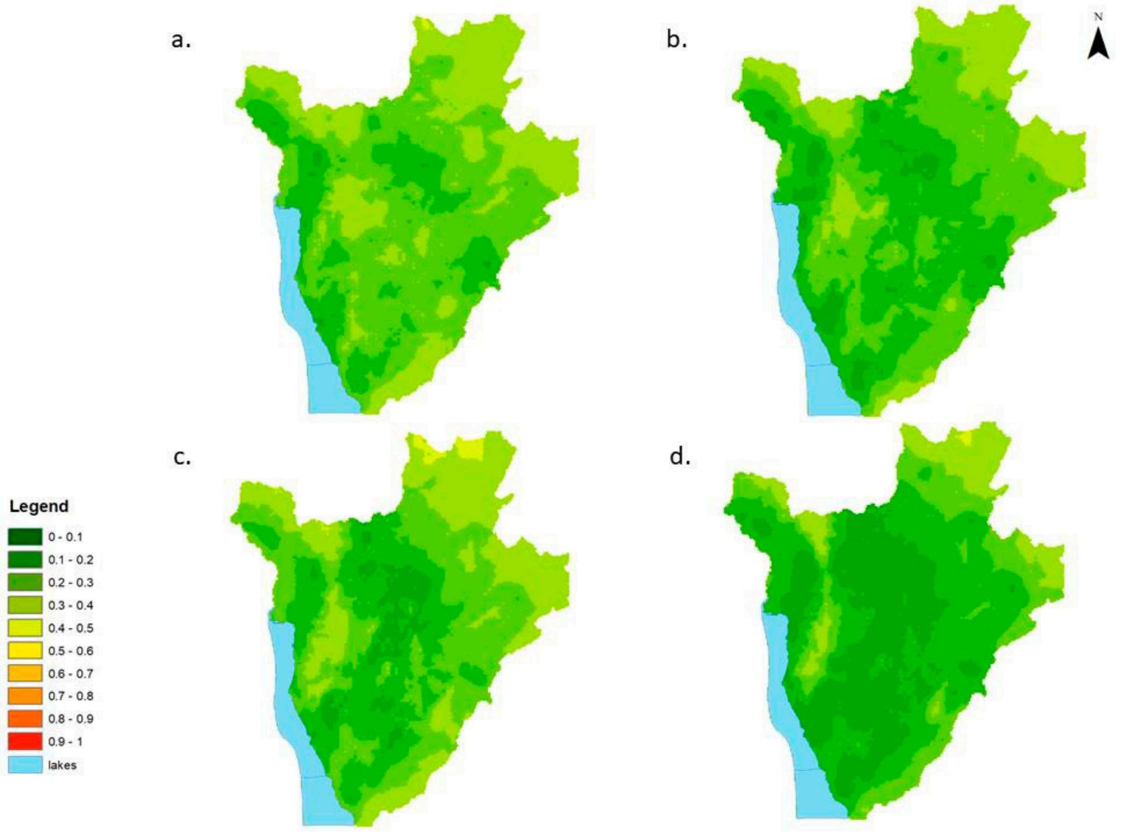


The intensity of the colour represents the magnitude of the standard deviation (see accompanying legend) for the proportion of school-aged children with moderate- and high-severity anaemia (see Table 1 of main text for classification of anaemia and anaemia severity categories according to the World Health Organization [1]). The pale blue colour depicts the location of Lake Tanganyika. **(a)** 2008, **(b)** 2009, **(c)** 2010, **(d)** 2011.

**Annex Reference**

1. World Health Organization. Iron deficiency anaemia: assessment, prevention and control. A guide for programme managers. 2001; WHO/NHD/01.3. Available: http://www.who.int/vmnis/indicators/haemoglobin.pdf (accessed 30 May 2018).
